# Optimizing inference of segmentation on high-resolution images in MLExchange

**DOI:** 10.1007/s11227-025-07413-5

**Published:** 2025-06-20

**Authors:** Shizhao Lu, Tanny Chavez, Wiebke Koepp, Guanhua Hao, Petrus H. Zwart, Alexander Hexemer

**Affiliations:** 1 Advanced Light Source, Lawrence Berkeley National Laboratory, Berkeley, CA 94720 USA; 2 Center for Advanced Mathematics for Energy Research Applications, Lawrence Berkeley National Laboratory, Berkeley, CA 94720 USA; 3 Molecular Biophysics and Integrated Bioimaging Division, Lawrence Berkeley National Laboratory, Berkeley, CA 94720 USA; 4 Berkeley Synchrotron Infrared Structural Biology Program, Lawrence Berkeley National Laboratory, Berkeley, CA 94720 USA

**Keywords:** Image segmentation, Model inference, Distributed computing, Multi-GPU, Machine learning, Tomography

## Abstract

MLExchange is a machine learning (ML) operations platform providing web user-interfaces (UIs) for data visualization and analysis pipelines at synchrotron facilities. Among these UIs is the segmentation app which helps synchrotron users utilize ML algorithms to automatically segment high-resolution scientific images with minimal manual annotation effort. In this work, we share code optimizations that significantly speed up the segmentation inference workflow of large data in short time. By optimizing the sequence of CPU-GPU data transfers and introducing CPU parallelization to key operations, we improve the per-device, per-image frame computational efficiency and observe close to 3$\times$ speedup over the original segmentation inference workflow run time when utilizing a single GPU. Further adaptations enabling multi-GPU inference yield more than 40$\times$ speedup with 100 GPUs compared to the optimized single GPU inference workflow. This acceleration of the segmentation inference workflow will provide MLExchange users with easy access to segmentation results with little wait time.

## Introduction

The U.S. Department of Energy (DOE) scientific user facilities (SUFs) are leading the generation of scientific data through a large collection of advanced experimental facilities, ranging from particle accelerators, light sources, and neutron scattering sources. Artificial intelligence (AI) has shown great potential at accelerating discovery and synthesis of novel materials [1, 2].

The Frontiers in AI for Science, Security, and Technology (FASST) initiative has been recently proposed by the DOE to establish infrastructure for building integrated scientific AI systems and platforms [3]. It aims to integrate classified and unclassified scientific data into a large, AI-ready repository for training, testing, development, and validation of scientific AI models, and to support and accelerate the development of AI models across various domains of the physical sciences [3]. FASST aligns well with the initiatives for more powerful computational resources, such as the Integrated Research Infrastructure (IRI) [4] and the high performance data facility (HPDF) hub [5] in the DOE system as well as completed and upcoming infrastructure upgrades at multiple SUFs [6, 7].

The Advanced Light Source, a SUF at Lawrence Berkeley National Laboratory (LBNL), is going through a major upgrade known as the ALS-U. The upgrade will bring 20$\times$ increase of the beam current and 100$\times$ increase of the beam brightness. [6] The rates of data generation for several beamlines are expected to increase by many folds. An AI@ALS workshop [8] highlighted recent and ongoing efforts in development and integration of AI and ML at the accelerator, beamlines, and data analysis workflows. One of the challenges is lowering the barrier for users at the beamline to use complex ML algorithms without much personal investment in learning the techniques.

MLExchange [9] is a machine learning (ML) operations platform that hosts and deploys web-based user-interfaces (UIs) for data ingestion, visualization, and analysis pipelines at SUFs. This platform is a cross-facility collaborative effort among five U.S. DOE national laboratories: Lawrence Berkeley National Laboratory (LBNL), Brookhaven National Laboratory (BNL), Argonne National Laboratory (ANL), Oak Ridge National Laboratory (ORNL), and SLAC National Accelerator Laboratory. The MLExchange platform makes complex ML models available to synchrotron users for easy access and integration with data analysis pipelines.

**Fig. 1:**
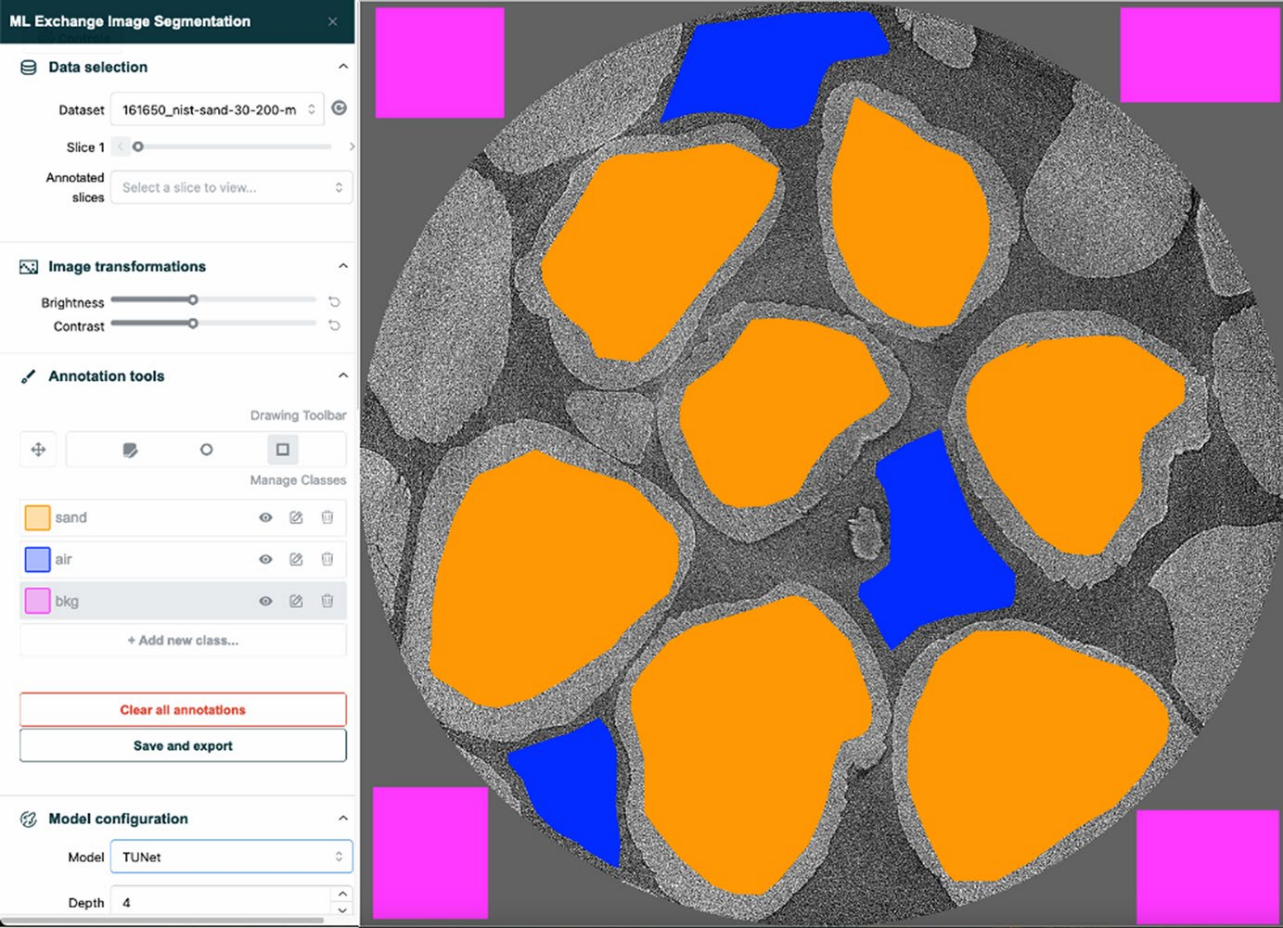
Snapshot of the MLExchange image Segmentation App where a user has created manual segmentation annotations overlaying a selected slice of a X-ray tomography dataset containing sand grains. Aside from creating different annotation classes and shapes, the UI supports navigation of the dataset; basic image transformations with adjustment to brightness, and contrast; loading and saving of created annotations; as well as options to tune the configuration of ML models to be used for training and inference

One example is the high-resolution image-segmentation application (Segmentation App) that enables users to intuitively annotate high-resolution scientific image data on a web-based UI and use these annotations to train and perform inference with ML-based segmentation models. This application makes use of the deep learning for scientific image analysis (DLSIA) [10] Python library, that provides customizable neural network architectures for various machine learning data analysis tasks, to enable ML-based segmentation.

In addition, MLExchange uses Tiled [11] from the Bluesky [12] scientific data management ecosystem for data access, and Prefect [13] for workflow orchestration. The Segmentation App has been successfully applied for various use cases [14]. Aside from the high-resolution reconstructed tomography images that are subject of this work, further use cases are the segmentation of powder X-ray diffraction images [15] and microscopy images [16].

**Fig. 2:**
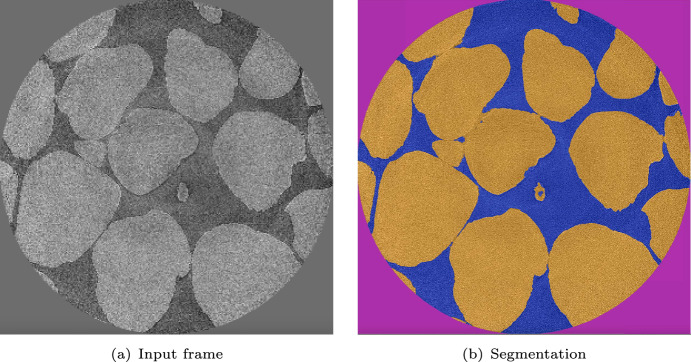
Example of a single frame of the X-ray tomography characterization of sand grains and its segmentation

The general layout of the Segmentation App UI is shown in Fig. 1. This interface displays a single frame from a tomography data set of sand grains, collected at ALS Beamline 8.3.2 in white-light mode, with manually created class annotations consisting of three classes: sand, air, and background. The control panel on the left-hand side contains parameters for data selection, image transformations, annotation tools, and model configuration. The segmentation result for this data set is displayed in Fig. 2.

With the anticipation of ALS-U, we are also expecting increase in the scientific image data throughput from several beamlines at the ALS [8, 17]. To keep pace with the elevated need for faster, on-the-fly scientific image data processing, we have to reevaluate and increase the computational efficiency of our segmentation inference backend of the Segmentation App. In this paper, we present run time optimizations for the segmentation inference workflow in the MLExchange platform. We describe the original and the optimized workflows in Sect. 3 and show the speed up results in Sect. 4. We conclude and give directions for future work in Sect. 5.

## Background

Here, we briefly describe the software packages that we used in optimizing the segmentation inference workflow in MLExchange.

**Deep Learning for Scientific Image Analysis (DLSIA)**: DLSIA [10] contains a collection of ML models such as mixed-scale dense convolutional neural networks (MSDNets) [18], Tunable U-Nets (TU-Nets) [19], Tunable U-Net3+ (TU-Net3+) [20], and randomized Sparse Mixed-Scale networks (SMSNet) built in PyTorch for scientific image analysis.

**Numba**: Numba [21] is an open-source Just-In-Time (JIT) compiler package that translates Numpy and some parts of Python code into machine code for fast execution. Numba has options for CPU and GPU parallelization.

**NVIDIA NSight Systems**: NVIDIA NSight Systems [22] is a profiler for analysis and visualization of code run time and CPU/GPU activity. It supports user-defined profiling annotations through the NVIDIA Tools Extension Library (NVTX), enabling detailed profiling of specific code regions. We make use of Pytorch’s NVTX integration [23].

**PyTorch Distributed**: PyTorch Distributed [24] is a Torch package for supporting multiprocess parallelization and communication across multiple GPU cores and nodes.

**qlty**: qlty [25] is a tensor handling toolkit for efficiently cropping high-resolution scientific images that otherwise would not fit into GPU memory into smaller images. When applied in a segmentation workflow, it uses overlapping regions to perform ensemble-style aggregation, producing a seamless and coherent full image prediction.

**Fig. 3:**
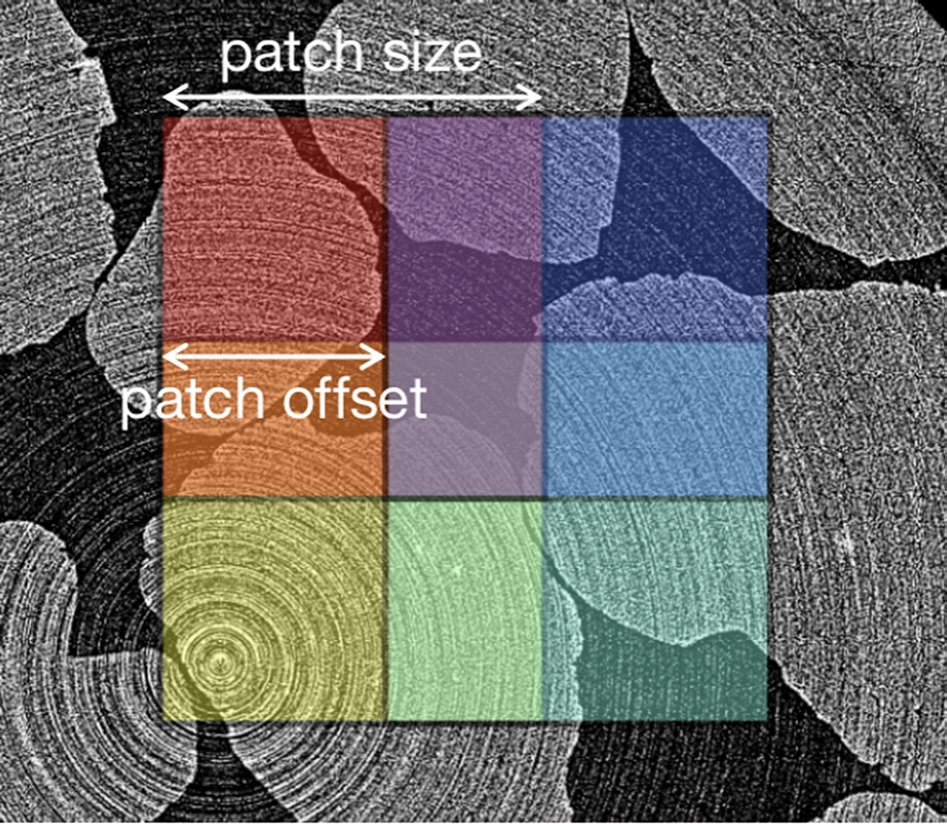
Snapshot of a cropped image with four qlty patches of patch size 512 and patch offset 300

Figure 3 shows an example of the qlty patches overlaying the sand tomography image. Segmentation accuracy improvements can occur through the ensemble approach depending on the extent of effective overlap between patches, which is jointly determined by the patch size, the step size, and the blending weights. Increasing overlap can improve accuracy but also incurs additional processing time due to the larger number of patch evaluations and blending operations.

## Methods

The Segmentation App is a Python-based web UI for ML-based segmentation of high-resolution scientific images hosted in the MLExchange platform. It has a front-end interface for users to load image data from a given Tiled server and intuitively create manual annotations for one or multiple frames of the image dataset. Built within the Bluesky ecosystem [12], Tiled [11] enables structured, sliceable access to diverse data formats from filesystems and databases, making it well suited for serving scientific imaging data to the app. Users can then submit a job to a Prefect [13] server to train a segmentation model through the DLSIA [10] framework on the annotated image frames. Once training completes, the partial segmentation of these annotated frames is visualized in the UI for the user’s review. After inspection of the partially segmented results, users can submit a follow-up job to the prefect server to run segmentation inference on the full data stack, which includes the frames that were not annotated.

The segmentation training and inference Python scripts are called in the backend when the prefect server orchestrates either of these jobs. Because the trained models can be used not only for the dataset it was trained on, but also for similar image data, users will likely run more inference jobs than training jobs. This is also true for users with large-scale high-resolution scientific data that often comes with thousands of frames in one experiment. To further accelerate this process, pre-trained models can also be hosted on a shared public server in the future, allowing users to perform inference directly in the Segmentation App without needing to train their own models.

For both the training and inference of the segmentation models, we have used the qlty package [25] to crop the high-resolution scientific images into smaller overlapping patches. Segmentation is then performed on those small image patches to avoid out-of-memory issues. In addition, the overlap among cropped patches enhances the segmentation results by averaging the overlapping predictions along the borders of the patches, resulting in a partial ensemble approach.

Although our training and inference workflows are currently done on local machines and edge servers, we also have the capability to run them on supercomputing clusters. Our vision for the Segmentation App moving forward is for users to have easy access to a variety of computing resources where they can submit inference jobs and obtain the segmentation results quickly.

In our current study, we focus on optimizing the inference workflow. In this section, we first describe our original segmentation inference workflow, previously developed for running on a single GPU. We then describe additional optimizations to the workflow for running in a multi-GPU configuration.

### Original workflow

Here, we present the original segmentation inference workflow and give a brief description of the functionality of each operation in the workflow.

Our original segmentation inference workflow can be broken down into eight operations: Normalize Data, qlty Unstitch, CPU-to-GPU Data-Copy, Inference, GPU-to-CPU Data-Copy, Torch Concat, qlty Stitch, and Torch Argmax.

The input image data is first normalized by clipping the intensity values between 1% and 99% percentiles during Normalize Data to remove outlier intensities.

Instead of using the full image as input, which would be computationally and memory intensive, we first crop the full image into many smaller patches to use as input for the segmentation model.

Qlty Unstitch is a function from qlty [25], that crops the input image data into smaller images defined by the qlty patch size (dimensions of the cropped images in pixels) and the qlty patch offset (pixel shift between consecutive patches, which determines the degree of overlap).

The qlty patches are then divided into batches and for each batch of these smaller images, we copy the data from CPU to GPU. Inference is then performed with the trained segmentation model, and the predicted class probabilities of each pixel are copied back from the GPU to CPU.

We then concatenate all the qlty patches into a single multi-dimensional tensor with dimensions $\#$ patches $\times$ patch size $\times$ patch size. This concatenated tensor is passed through the qlty stitch function to reconstruct the full image’s predicted class probabilities using a weighted average.

Finally, we use Torch Argmax to obtain the predicted class labels of each pixel within the full image.

### Optimized workflow

To keep up with the demand of on-the-fly segmentation of large volume scientific image data, we want to increase the time efficiency of our segmentation inference workflow. Using NVIDIA Nsight Systems profiler and making use of PyTorch’s NVTX integration [23], we have identified several parts in the original workflow that may benefit either from moving the operation to GPU, bulk data copy, or introducing parallelization techniques.

**Fig. 4:**
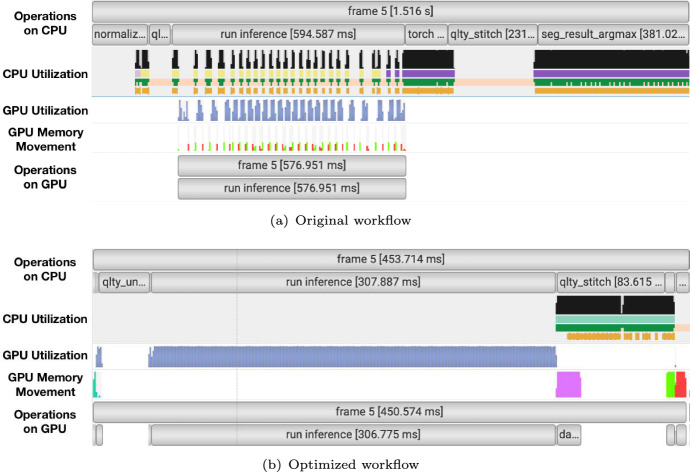
NVIDIA Nsight Systems profiles of one image frame using the (**a**) original and (**b**) optimized workflow. The labels on rows correspond to NVIDIA Tools Extension Library (NVTX) time tracker labels we assigned for different operations with torch.cuda.nvtx.range_push(...) and torch.cuda.nvtx.range_pop() [23]. Qlty patches are of patch size of 64 and patch offset of 32. Note that time scales are not normalized across (**a**) and (**b**) in order to preserve the original profiling context and highlight structural differences in GPU execution behavior.

In Fig. 4a, we notice that the inference operation takes less than half of the run time of the segmentation inference workflow; much time is spent on performing tensor operations on CPU such as Normalize Data, Torch Concat, and Torch Argmax. We also notice that because data movement occurs at every batch, the GPU utilization is interrupted with periods of low utilization throughout the Inference operation.

**Fig. 5:**
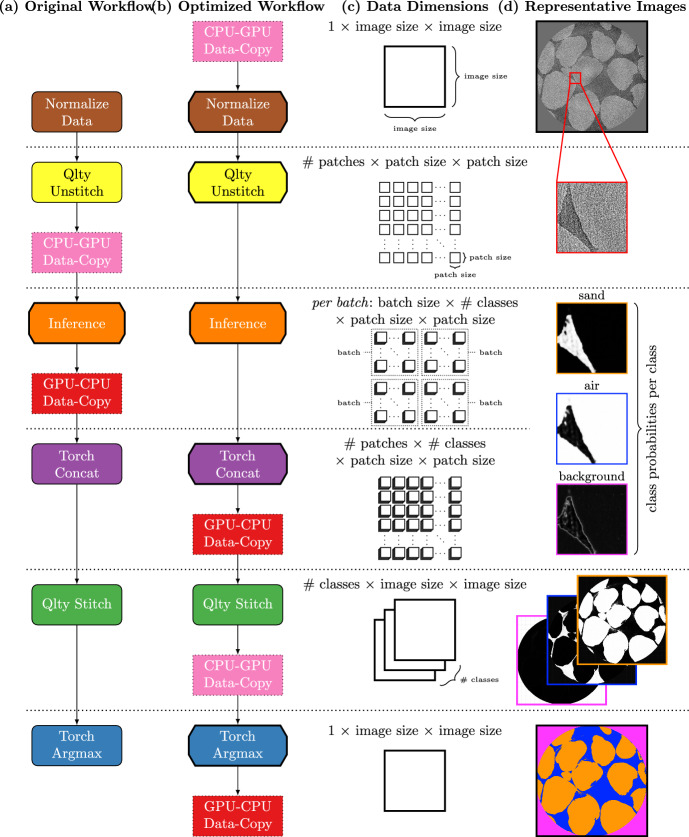
Flowcharts of (**a**) the original segmentation inference workflow, and (**b**) the optimized segmentation workflow. Rectangle boxes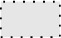 are data copy operations, rounded rectangle boxes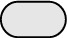 indicate operations performed on CPU, chamfered boxes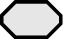 indicate operations performed on GPU. (**c**) Schematics of data transformations and their dimensions (**d**) Examples of intermediate results

To improve the performance of the operations in the original workflow mentioned above, we redesign the segmentation inference workflow to optimize data copy and moving tensor operations on GPU. The original and the optimized workflow is shown in two separate flowcharts in Fig. 5.

In the optimized workflow, we first copy the full image data from CPU to GPU in bulk. Afterward, all the operations can be done on GPU with the exception of qlty Stitch which does not benefit from moving the operation to GPU. Instead, we develop a Numba [21] Just-In-Time (JIT) implementation of the qlty Stitch function that also utilizes parallelization of CPU processes.

The outcome of the optimization can be seen in Fig. 4b. The total run time is reduced to about one third of the original workflow. The three tensor operations Normalize Data, Torch Concat and Torch Argmax are no longer taking significant run time when performed on GPU. The inference operation is also reduced in run time thanks to uninterrupted high GPU utilization. Note that the experiments in this study were run on a supercomputer with large RAM (40 GB High Bandwidth Memory (HBM) per GPU) to benefit from the speedup gained from bulk data copy. Memory should be taken into consideration when deciding between batched or bulk data copy between devices.

## Results and discussion

**Fig. 6:**
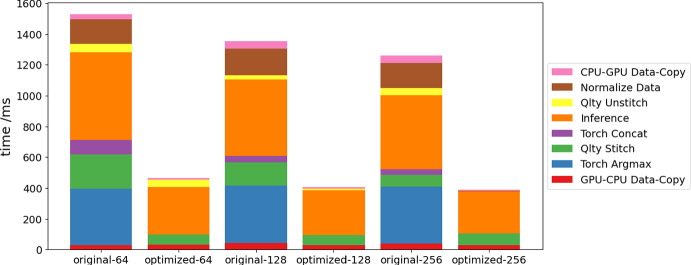
Breakdown of segmentation inference workflow run time using the original workflow and the optimized workflow on a single GPU device, averaged over 10 image frames, where 64, 128 and 256 correspond to the qlty patch size configurations used for each analysis

**Table 1:** Breakdown of segmentation inference workflow run time using the original workflow and the optimized workflow on a single GPU device, averaged over 10 image frames, where 64, 128 and 256 correspond to the qlty patch size configurations used for each analysis

Operation Run Time [ms]	Orig 64	Opt 64	Orig 128	Opt 128	Orig 256	Opt 256
CPU-GPU Data-Copy	29.75 ± 6.62	8.56 ± 4.76	48.49 ± 0.12	8.55 ± 4.76	49.29 ± 22.20	8.42 ± 4.62
Normalize Data	161.57 ± 10.21	1.06 ± 0.05	172.43 ± 11.22	1.04 ± 0.02	163.43 ± 8.47	1.04 ± 0.01
Qlty Unstitch	53.67 ± 2.63	46.54 ± 2.55	29.94 ± 0.90	11.40 ± 0.19	44.92 ± 44.50	2.72 ± 0.07
Inference	567.81 ± 52.19	308.46 ± 0.09	493.81 ± 27.94	289.32 ± 0.23	481.48 ± 39.89	271.94 ± 0.20
Torch Concat	96.32 ± 8.69	0.07 ± 0.01	43.27 ± 5.54	0.06 ± 0.03	33.61 ± 3.48	0.06 ± 0.02
Qlty Stitch	219.30 ± 4.33	68.28 ± 9.83	149.26 ± 20.02	65.48 ± 10.44	78.22 ± 11.66	77.47 ± 13.84
Torch Argmax	369.92 ± 13.46	0.10 ± 0.01	373.40 ± 11.97	0.10 ± 0.01	369.28 ± 7.58	0.09 ± 0.00
GPU-CPU Data-Copy	27.50 ± 11.08	30.98 ± 16.17	43.05 ± 2.36	29.35 ± 16.39	39.76 ± 17.41	27.94 ± 16.28

Reported values represent the average and standard deviation

For our experiments in this study, we use an X-ray tomography dataset of sand particles characterized at ALS Beamline 8.3.2, shown in Figs. 1 and 2. The full stack of images consists of 2160 images of size 2560x2560 (52.7GB in disk storage space). We refer to each image in the stack as a frame.

We use NumPy memory-mapping strategies to load a single frame of image data from the NumPy array of the full tomography image stack saved locally, instead of loading the entire dataset into memory. Memory-mapped arrays help mitigate the memory footprint of the processed data, especially when we are not processing the entire data at the same time and on the same computing device. However, this memory-mapping option is great for offline data processing when you have all your data saved, but may not be available or feasible for cases when we want to process data on-the-fly.

We made annotations consisting of three label classes: sand, air, and background with the Segmentation App in the MLExchange platform for one image frame. The image frame and its annotations are used to train a tunable UNet (TUNet) [19] implemented for the analysis of scientific images [10] with the following model specifications: depth of 4, 32 base channels, growth rate of 2, hidden rate of 1, 30 epochs, Adam optimizer [26], CrossEntropy loss function with class weight of 1 for all three label classes, learning rate of 0.001, ReLU activation function [27], and 32 training batch size.

### Optimization-single frame

We run scaling studies on the GPU nodes of the NERSC Perlmutter [28] supercomputer, each with a third-generation AMD EPYC 7763 CPU (128 threads per CPU) with 256 GB of DDR4 DRAM and four NVIDIA A100 GPUs (40 GB HBM per GPU). In our segmentation inference runs, we use 32 CPU threads per GPU and an inference batch size of 256.

We present the runtime data obtained using the NVIDIA Nsight Systems profiler as bar plots in Fig. 6 and in tabular form in Table 1. These compare the performance of the original and optimized segmentation inference workflows, using different qlty patch configurations (detailed in Table 2), across 10 image frames processed on a single GPU.

**Table 2:** Qlty Patch Configurations

Patch size	Patch offset	Number of patches
64	32	6241
128	64	1521
256	128	361

The run time of Normalize Data, Torch Concat and Torch Argmax reduced from 161.57–172.42 ms to 1.04-1.06 ms, 33.61–96.32 ms to 0.06-0.07 ms, and 369.28–373.40 ms to 0.09-0.10 ms, respectively, using the optimized workflow. For other operations, we observe some degrees of reduction in run time though not as drastic as the three tensor operations. Inference operation run time is reduced by 40–45% from freeing up data copy time and reduced GPU idle time. We note that the segmentation inference results using the optimized workflow are identical to that using the original workflow.

**Fig. 7:**
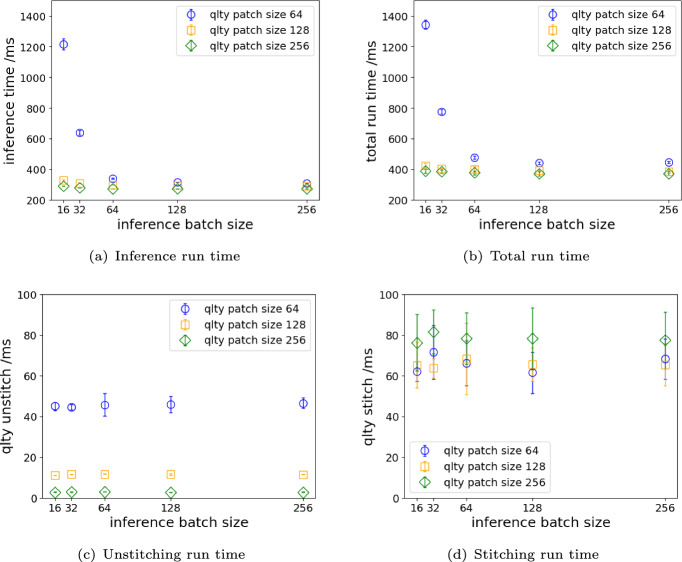
Impact of the inference batch size on the run time of (**a**) the inference operation, (**b**) the entire workflow, (**c**) unstitching and (**d**) stitching with qlty in the optimized segmentation workflow, averaged over 10 image frames

For the qlty Stitch operation, we see the largest speedup for qlty patch size 64 indicating that the speedup efficiency of our Numba implementation on CPU increases with the number of qlty patches. For the qlty Unstitch operation however, the speedup increases with qlty patch size when operated on GPU in the optimized workflow compared to on CPU in the original workflow.

Additionally, we studied the impact of the inference batch size on the run time of the optimized segmentation inference workflow with three qlty patch configurations.

Figure 7a presents a sharp decrease in inference run time for a qlty patch size of 64 as the inference batch size increases from low to high. However, for the other qlty patch sizes 128 and 256, the run times of the inference operation are not as affected by the batch size because there are fewer number of patches compared to the case of qlty patch size of 64.

We see the same trend in the total run time of the optimized segmentation inference workflow in Fig. 7b as the inference operation is now the majority component of the workflow after optimization.

For qlty Unstitch and qlty stitch shown in Fig. 7c and d, we don’t observe dependence of the run time of the two qlty operations on the inference batch size. The differing runtime trends for stitch and unstitch, as seen in Fig. 7c and d, arise from how each operation is executed. Unstitch uses pure tensor slicing and runs entirely on the device hosting the input tensor, in our case the GPU(s). Larger patches mean fewer slices, resulting in faster execution. Stitch, on the other hand, is implemented as a Numba-accelerated CPU kernel and is primarily constrained by memory bandwidth. While its overall runtime is largely stable across patch sizes, very large tiles introduce subtle penalties due to reduced thread utilization and increased memory traffic, resulting in a modest but noticeable increase in run time.

Note that we do not show the run time of Normalize Data, Torch Concat and Torch Argmax operations as they become negligible when done on GPU as shown in Table 1.

### Optimization-whole stack

**Fig. 8:**
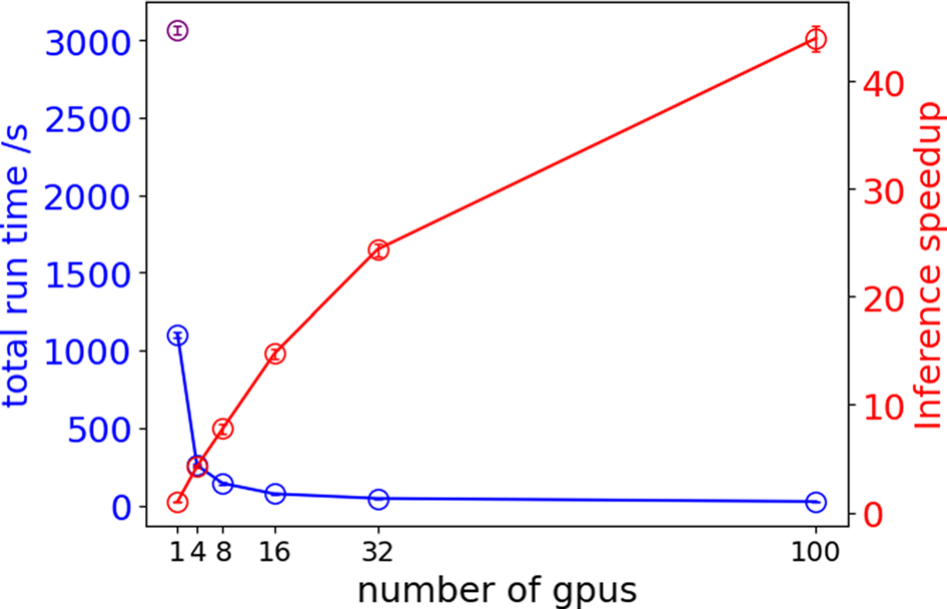
Scaling analysis of the optimized segmentation inference workflow run time, qlty patch size of 64. Segmentation inference workflow run time of the optimized workflow is plotted in blue, the purple dot is the segmentation inference workflow run time of the original code with a single GPU plotted for reference. Segmentation inference workflow run time speed up is plotted in red. Error bars represent the standard deviation calculated over five independent runs; note that initialization time is included in total runtime measurements but excluded from inference time

In Fig. 8 and Table 3, we show the result of the multi-GPU speedup using the Torch Distributed package [24] when performing segmentation on the whole stack of tomography images (2160 frames).

**Table 3:** Segmentation inference workflow run time when using multi-GPU implementation with optimized code and speedup ratio compared to the original code ran on a single GPU and the optimized workflow on a single GPU for a whole stack of tomography images

Number of GPUs	1	4	8	16	32	100	Original
Total Run Time/s	1068.97	259.07	142.44	74.95	45.28	24.34	3065.70
Speedup (Original)	2.87	11.83	21.52	40.90	67.70	125.96	1
Speedup (Opt 1 gpu)	1	4.13	7.50	14.26	23.61	43.92	N/A

Listed are averages over five independent runs

**Table 4:** Initialization times when using multi-GPU implementation with optimized code and with the original code ran on a single GPU

Number of GPUs	1	4	8	16	32	100	Original
Initiate Device/s	13.24	17.33	20.57	15.11	13.83	18.09	12.49
Load Modules/s	13.15	12.84	12.95	12.88	13.33	12.87	13.06
Initiate Torch Distributed/s	0.24	3.46	3.54	3.95	4.66	4.19	0.26
Load Data/s	0.4	0.10	0.10	0.11	0.10	0.11	0.49

Listed are averages over five independent runs

The purple dot shows the total run time using the original segmentation inference workflow (single GPU) for processing 2160 image frames, which corresponds to approximately

Our optimized workflow obtained a 2.87$\times$ speedup compared to the run time of the original segmentation workflow on a single GPU before using the optimization methods we described in the paper. Furthermore, the multi-GPU strategy presented in this manuscript achieved a 40$\times$ speedup relative to the optimized single GPU inference workflow. We note that on Perlmutter (and other supercomputing facilities), job queue wait times pose a practical constraint for short inference tasks, particularly those completing in under five minutes. In our study, we observed near-linear performance scaling up to 16 GPUs. However, beyond this point, the gains diminish due to increased inter-node and inter-core communication and parallel overhead. Moreover, jobs requesting large GPU counts (e.g., 100 GPUs) often experienced queue times significantly longer than the actual runtime, which is inefficient for users requiring low-latency inference, e.g. for on-the-fly analysis during beamtime experiments. From a resource management perspective, such configurations may also result in poor allocation utilization, as large GPU requests tied to short jobs may lead to underutilized reserved time slots. These findings underscore the importance of balancing performance with resource efficiency, and suggest that dedicated short-job queues could greatly enhance the effectiveness of our optimized segmentation inference workflow on HPC systems.

In Table 4 we also show the initialization time when running our workflow on Perlmutter’s exclusive GPU nodes. Aside from the additional 3–4 s required to initiate the Torch Distributed setup for multi-GPU runs, the overall initialization time increases by 4–5 s across workflow runs with varying numbers of GPUs, compared to the single GPU run.

Single GPU pipelines may encounter performance bottlenecks under high-throughput workloads, such as memory saturation during large-batch processing [29], which can degrade inference speed when scaling to data-intensive applications like tomography. The proposed optimization strategies were designed to address these limitations by leveraging parallelism and batching to improve throughput and scalability. However, it is also important to recognize that the effectiveness of the proposed optimization strategies is subject to system-level constraints. For instance, batched data transfer is limited by available GPU memory, which can restrict batch sizes and reduce scalability for large datasets. In our experiments, we mitigated these limitations by utilizing a supercomputing environment with ample GPU memory; however, on memory-constrained systems, the performance gains from such optimizations may be less pronounced.

Finally, it is important to note that this work focuses exclusively on inference, where gradient and weight synchronization are not required. As such, our emphasis is on efficient data distribution and result aggregation across devices. In future work, we plan to extend our approach to training scenarios, enabling a more comprehensive evaluation of multi-GPU scaling strategies.

## Conclusion and future work

We have identified bottlenecks in the original segmentation inference code by using performance analysis tools. We have optimized the segmentation inference workflow by rearranging the sequence of data movement and reassigning workflow operations at the device (either CPU or GPU) that can better reduce the run time. This paper introduced a multi-GPU parallelization strategy based on the Torch Distributed package to further speed up the inference run time. Overall, the multi-GPU enabled segmentation inference workflow achieved more than a 40$\times$ speedup compared to the optimized single GPU version. By improving the performance and scalability of the segmentation inference workflow, we enable users of our MLExchange platform to get segmentation results of large-scale experiments in a short amount of time.

Future developments of the segmentation pipeline include run time performance optimization at training and deployment. We will also explore integration with Tiled data services into the segmentation inference workflow that runs on supercomputers. As part of these efforts, we aim to investigate performance portability and tuning strategies across diverse GPU architectures, to ensure robust acceleration on a broader range of computing platforms.

## References

[1] Szymanski NJ, Rendy B, Fei Y, Kumar RE, He T, Milsted D, McDermott MJ, Gallant M, Cubuk ED, Merchant A, Kim H, Jain A, Bartel CJ, Persson K, Zeng Y, Ceder G (2023) An autonomous laboratory for the accelerated synthesis of novel materials. Nature 624(7990):86–91. 10.1038/s41586-023-06734-w 38030721 10.1038/s41586-023-06734-w PMC10700133

[2] Merchant A, Batzner S, Schoenholz SS, Aykol M, Cheon G, Cubuk ED (2023) Scaling deep learning for materials discovery. Nature 624(7990):80–85. 10.1038/s41586-023-06735-9 38030720 10.1038/s41586-023-06735-9 PMC10700131

[3] DOE: Frontiers in Artificial Intelligence for Science, Security and Technology (FASST). Accessed: 2024-10-27 (2024). https://www.energy.gov/fasst

[4] Miller WL, Bard D, Boehnlein A, Fagnan K, Guok C, Lançon E, Ramprakash SJ, Shankar M, Schwarz N, Brown BL (2023) Integrated Research Infrastructure Architecture Blueprint Activity (Final Report 2023). OSTI 1984466, US DOE Office of Science (SC). 10.2172/1984466

[5] DOE: High Performance Data Facility: Supporting the Data Life Cycle. Accessed: 2024-10-27 (2024). https://hpdf.science/

[6] Advanced Light Source (ALS): ALS-U Timeline and Facility Impacts. Accessed: 2024-09-26 (2024). https://als.lbl.gov/als-u/als-u-timeline/

[7] Borland M, Abliz M, Arnold N, Berenc T, Blednykh A, Byrd J, Calvey J, Carter J, Carwardine J, Cease H, Conway Z, Decker G, Dooling J, Emery L, Fuerst J, Harkay K, Jain A, Jaski M, Kallakuri P, Kelly M, Kim S-H, Lill R, Lindberg R, Liu J, Liu Z, Nudell J, Preissner C, Sajaev V, Sereno N, Sun X, Sun Y, Veseli S, Wang J, Wienands U, Xiao A, Yao C (2018) The Upgrade of the Advanced Photon Source. In: Proceedings of the 9th International Particle Accelerator Conference, pp. 2872–2877. 10.18429/JACOW-IPAC2018-THXGBD1

[8] Parkinson DY, Chavez T, Choudhary M, English D, Hao G, Hellert T, Leemann SC, Nemsak S, Rotenberg E, Taylor AL, Scholl A, White AA, Islegen-Wojdyla A, Zwart PH, Hexemer A (2024) AI@ALS workshop report: machine learning needs at the advanced light source. Synchrotron Radiat News 37:49–64. 10.1080/08940886.2024.2391258

[9] Zhao Z, Chavez T, Holman EA, Hao G, Green A, Krishnan H, McReynolds D, Pandolfi RJ, Roberts EJ, Zwart PH, Yanxon H, Schwarz N, Sankaranarayanan S, Kalinin SV, Mehta A, Campbell SI, Hexemer A (2022) MLExchange: A web-based platform enabling exchangeable machine learning workflows for scientific studies. In: Proceedings of the 4th Annual Workshop on Extreme-scale Experiment-in-the-Loop Computing (XLOOP), pp. 10–15. 10.1109/xloop56614.2022.00007 10.1109/xloop56614.2022.00007 PMC10733127 38131031

[10] Roberts EJ, Chavez T, Hexemer A, Zwart PH (2024) DLSIA: deep learning for scientific image analysis. J Appl Crystallogr 57:392–402 38596727 10.1107/S1600576724001390 PMC11001410

[11] Bluesky Collaboration: Tiled: API to structured data. Accessed: 2024-09-30 (2024). https://github.com/bluesky/tiled

[12] Allan D, Caswell T, Campbell S, Rakitin M (2019) Bluesky’s Ahead: a mlti-facility collaboration for an a la carte software project for data acquisition and management. Synchrotron Radiat News 32(3):19–22. 10.1080/08940886.2019.1608121

[13] PrefectHQ: Prefect: workflow orchestration framework. Accessed: 2024-09-30 (2024). https://github.com/PrefectHQ/prefect

[14] Hao G, Roberts EJ, Chavez T, Zhao Z, Holman EA, Yanxon H, Green A, Krishnan H, Ushizima D, McReynolds D, Schwarz N, Zwart PH, Hexemer A, Parkinson D (2023) Deploying machine learning based segmentation for scientific imaging analysis at synchrotron facilities. Electron Imaging 35(9):2901–2905. 10.2352/ei.2023.35.9.ipas-290 10.2352/ei.2023.35.9.ipas-290 PMC10735246 38130938

[15] Yanxon H, Roberts E, Parraga H, Weng J, Xu W, Ruett U, Hexemer A, Zwart P, Schwarz N (2023) Image segmentation using u-net architecture for powder x-ray diffraction images arXiv:2310.16186 [cs.LG] 10.1107/S160057752600754X PMC13549334 42640747

[16] Liu Y, Vasudevan RK, Kelley KP, Funakubo H, Ziatdinov M, Kalinin SV (2023) Learning the right channel in multimodal imaging: automated experiment in piezoresponse force microscopy. npj Comput Mater. 10.1038/s41524-023-00985-x

[17] Feggeler T, Levitan A, Marcus MA, Ohldag H, Shapiro DA (2023) Scanning transmission x-ray microscopy at the advanced light source. J Electron Spectrosc Relat Phenom 267:147381. 10.1016/j.elspec.2023.147381

[18] Pelt DM, Sethian JA (2017) A mixed-scale dense convolutional neural network for image analysis. Proc Natl Acad Sci 115(2):254–259. 10.1073/pnas.1715832114 29279403 10.1073/pnas.1715832114 PMC5777062

[19] Ronneberger O, Fischer P, Brox T (2015) U-Net: Convolutional Networks for Biomedical Image Segmentation. In: Medical Image Computing and Computer-Assisted Intervention (MICCAI), Part III. Lecture Notes in Computer Science, vol. 9351, pp. 234–241. 10.1007/978-3-319-24574-4_28

[20] Huang H, Lin L, Tong R, Hu H, Zhang Q, Iwamoto Y, Han X, Chen Y-W, Wu J (2020) UNet 3+: A Full-Scale Connected UNet for Medical Image Segmentation. In: Proceedings of the IEEE International Conference on Acoustics, Speech and Signal Processing (ICASSP), pp. 1055–1059.10.1109/icassp40776.2020.9053405

[21] Lam SK, Pitrou A, Seibert S (2015) Numba: a LLVM-based Python JIT compiler. In: Proceedings of the Second Workshop on the LLVM Compiler Infrastructure in HPC. SC15, pp. 1–6. 10.1145/2833157.2833162

[22] NVIDIA: NVIDIA NSight Systems. Accessed: 2024-10-01 (2024). https://developer.nvidia.com/nsight-systems

[23] PyTorch Core Team: torch.cuda.nvtx – PyTorch documentation. Accessed: 2025-05-01 (2024). https://pytorch.org/docs/stable/cuda.html#nvidia-tools-extension-nvtx

[24] Li S, Zhao Y, Varma R, Salpekar O, Noordhuis P, Li T, Paszke A, Smith J, Vaughan B, Damania P, Chintala S (2020) PyTorch Distributed: Experiences on Accelerating Data Parallel Training. Proceedings of the VLDB Endowment 13(12), 3005–3018 10.14778/3415478.3415530arXiv:2006.15704 [cs.DC]

[25] Zwart PH (2024) Glty: handling large tensors in scientific imaging deep-learning workflows. Softw Impacts 21:100696. 10.1016/j.simpa.2024.100696 40206204 10.1016/j.simpa.2024.100696 PMC11981637

[26] Kingma DP, Ba J (2017) Adam: A method for stochastic optimization. 3rd International Conference on Learning Representations, ICLR 2015, Conference Track Proceedings arXiv:1412.6980 [cs.LG]

[27] Agarap AF (2019) Deep Learning using Rectified Linear Units (ReLU) arXiv:1803.08375 [cs.NE]

[28] National Energy Research Scientific Computing Center (NERSC): NERSC Perlmutter Architecture. Accessed: 2024-09-24 (2024). https://docs.nersc.gov/systems/perlmutter/architecture/

[29] Recasens PG, Agullo F, Zhu Y, Wang C, Lee EK, Tardieu O, Torres J, Berral JL (2025) Mind the memory gap: Unveiling gpu bottlenecks in large-batch llm inference arXiv:2503.08311 [cs.DC]

